# The Reversed Halo Sign in Pulmonary Infarction due to Acute Pulmonary Embolism

**DOI:** 10.5334/jbsr.3243

**Published:** 2023-09-07

**Authors:** Thalinne Schueremans, Margot Versavel, Adriana Dubbeldam

**Affiliations:** 1 KU Leuven, Belgium; 2 UZ Leuven, Belgium

**Keywords:** Atoll sign, reversed halo sign, RHS, pulmonary embolism, pulmonary infarction, HRCT, CT angiography

## Abstract

The reversed halo sign, or atoll sign, is a specific sign with ring-shaped consolidation and central lucency, which is historically considered typical for cryptogenic organising pneumonia. The presence of this sign in subpleural, posterior basal parts of the lower lobes, especially when solitary, should however raise suspicion for other causes, such as pulmonary infarction. Here, we present a case of pulmonary embolism with pulmonary infarction that was detected on HRCT without contrast.

**Teaching Point:** The presence of a reversed halo sign, especially when solitary and located in the periphery of the lower lobes, should raise suspicion of a pulmonary infarction.

## Introduction

The reversed halo sign (RHS), or atoll sign, is characterised as a central ground-glass opacity surrounded by a(n) (in)complete ring of consolidation on chest computed tomography (CT) [1,2,3,4]. Initially, it was considered specific for cryptogenic organizing pneumonia. However, the presence of RHS has since been reported in other diseases as well, including infectious (e.g., invasive fungal infections) and non-infectious causes (e.g., sarcoidosis, vasculitis, and pulmonary embolism [PE]).

Pulmonary infarction is a complication that affects around 10–30% of patients with an acute PE [2,3,4,5,6]. Although pulmonary infarction itself does not significantly affect the mortality rates [6], PE in general is correlated with high mortality rates up to 30%. The initial clinical symptoms of PE are usually non-specific and although pulmonary lab testing (D-dimers) can be helpful, they are also influenced by other causes. The diagnosis of PE is typically made on CT-angiography, yet a ventilation-perfusion scan can also be performed when available. Detecting possible pulmonary infarction on CTs without IV-contrast, can prompt further investigation with CT angiography in patients originally not suspected for pulmonary embolism, improving the patient outcome.

## Case History

**Figure 1:**
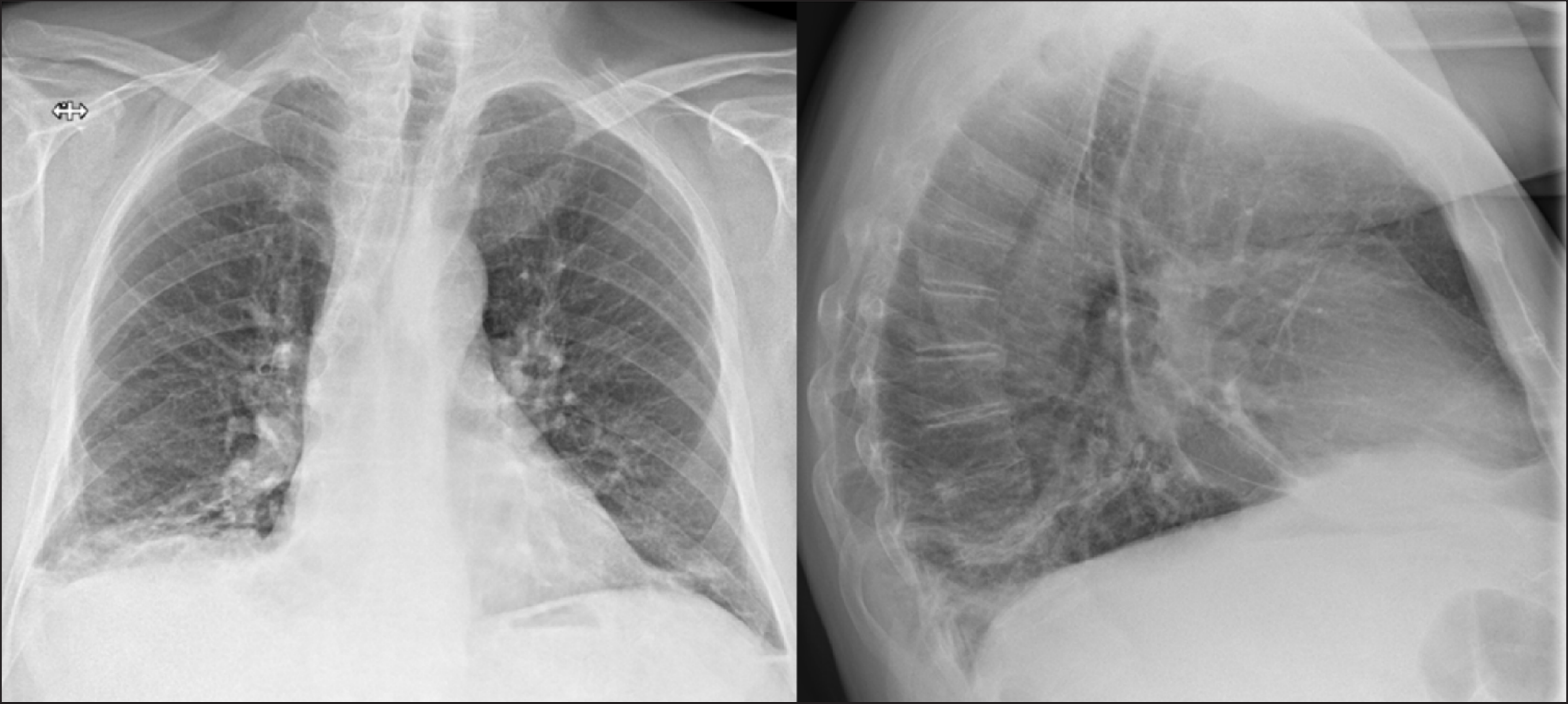
CXR with increased pleural fluid at the right lung basis and several dense strands.

An 80-year-old man with a prior history of chronic lymphocytic leukaemia, presented to the emergency room with recurring fever, dyspnoea, cough, and bibasilar crackles. Two weeks prior, he had been admitted for a right lower lobe (RLL) pneumonia. Further investigation showed high CRP (180 mg/L) and lymphocytic leucocytosis (115.000/µL). Initial chest X-ray (CXR) showed increased pleural fluid on the right and several dense strands (Figure 1). The patient was treated for pneumonia using antibiotics with a subsequent decrease in CRP.

**Figure 2:**
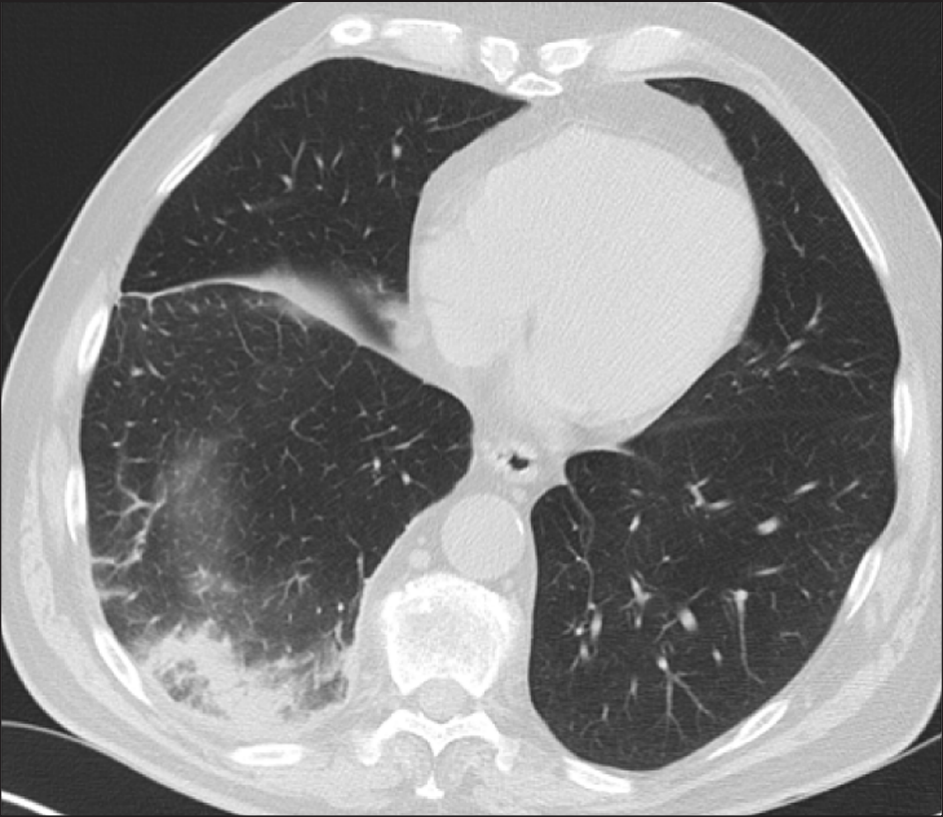
HRCT with a ground-glass opacity surrounded by a crescentic consolidated zone (RHS) in the subpleural space of the RLL.

**Figure 3:**
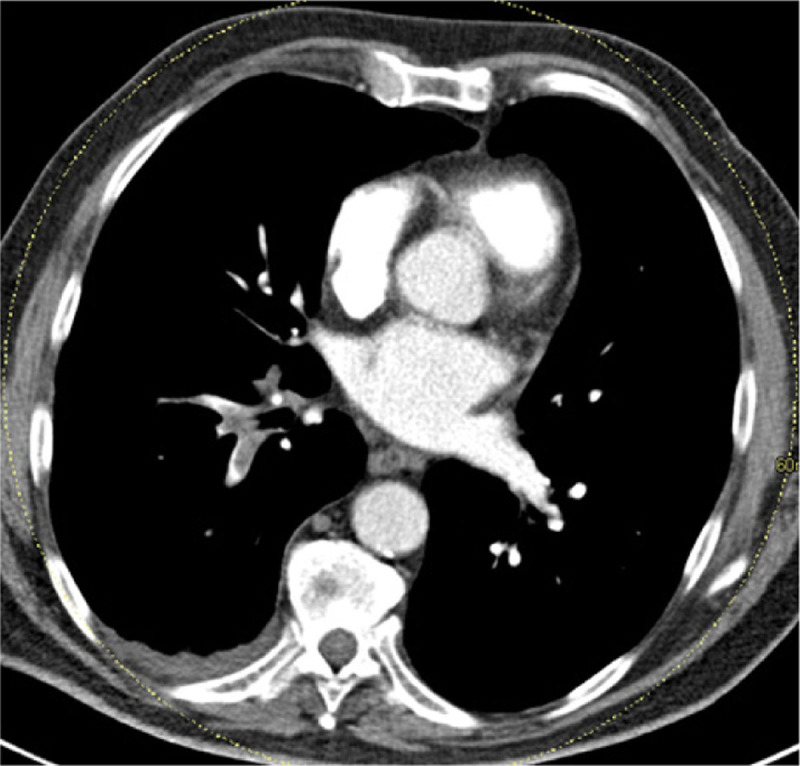
CT angiography with pathognomonic arterial filling defects.

However, during admission the cough persisted, the need for oxygen therapy increased, and the patient complained of right flank pain. A HRCT was performed which showed round consolidation, located subpleural in the dorsobasal part of the RLL accompanied by an atoll sign (Figure 2). Lung infarction was suspected by the radiologist and, after consulting the clinician, a CT angiography was performed. This showed (sub)segmental acute pulmonary thromboembolisms (Figure 3) and confirmed a lung infarction in the RLL. A low-molecular-weight heparin was started at therapeutic dose.

## Comments

This case signifies the RHS as an indirect parenchymal sign on plain CT for the diagnosis of pulmonary infarction, even in patients with no suspicion of PE. Although the RHS is historically suggestive for organising pneumonia, its localisation and distribution can suggest other diagnoses.

A retrospective study by Marchiori et al. [4] found multiple findings that can suggest a pulmonary infarction on plain CT: 1) the presence of internal areas of low attenuation; 2) located in the peripheral regions of the lower lobes; 3) most often single lesions and no more than two lesions; and 4) the presence of pleural effusion in half of the cases. They also report the importance of integrating clinical features: if the patient is immunocompromised, one must also suspect an invasive fungal infection. Others also found that these lesions are usually subpleural, in the posterior parts of the lower lobe, and affect the right lobe more often than left [3].

In retrospect, we can identify an enlarged right inferior hilum (Fleischner sign) with adjoining tapering of the pulmonary vessel (knuckle sign) on the initial CXR (Figure 1) [1,5]. These signs often coincide and are highly specific for PE, yet of low sensitivity. Other examples are the Westermark sign or Hampton-hump [1,5,6], whilst not clearly visible in this case. The initial diagnosis in our patient was delayed due to the recent history of pneumonia and since D-dimers were not measured. Luckily, when chest CT was finally performed, presence of the atoll sign in the subpleural, dorsobasal part of the RLL was quickly picked up as a possible lung infarction and CT angiography was advised, confirming the diagnosis.

## Conclusion

This case report shows that although the RHS or atoll sign is most commonly associated with cryptogenic organizing pneumonia, it is still important to consider differential diagnoses. A HRCT with a RHS, especially when solitary and/or in the dorsobasal parts of the lower lobes, should raise suspicion of a pulmonary infarction and should warrant further investigation, preferably with a CT pulmonary angiography.
